# USING NETWORK ANALYSIS METHODS TO STUDY MULTIMORBIDITY PATTERNS

**DOI:** 10.1093/geroni/igad104.2095

**Published:** 2023-12-21

**Authors:** Lauren Griffith, Alberto Brini, Edwin van den Heuvel, Philip St John, Lucy Stirland, Alexandra Mayhew, Graciela Muniz-Terrera

**Affiliations:** McMaster University, Hamilton, Ontario, Canada; Eindhoven University of Technology, Eindhoven, Noord-Brabant, Netherlands; Eindhoven University of Technology, Eindhoven, Noord-Brabant, Netherlands; Max Rady College of Medicine, Winnipeg, Manitoba, Canada; Global Brain Health Institute, San Francisco, California, United States; McMaster University, Hamilton, Ontario, Canada; Ohio University, Athens, Ohio, United States

## Abstract

Multimorbidity is a risk factor for patient-important outcomes including quality of life and functional decline. Multimorbidity research has focused mainly on disease counts, with less attention to patterns among chronic conditions. Network analysis has been increasingly used to examine multimorbidity clusters, but there are no guidelines for its conduct. In 12 recent studies using network analysis, we found heterogeneity in association measures (10 different measures) and clustering algorithms (5 different methods) used to identify multimorbidity clusters. Using self-reported data on 24 diseases in community-living adults aged 45-85 from the Canadian Longitudinal Study on Aging, we conducted network analyses using the 10 association measures and 5 clustering algorithms to better understand how these choices impact the number and types of clusters identified. We compared the similarity among clusters using the adjusted Rand index (ARI); an ARI of 0 is equivalent to the diseases being randomly assigned to clusters and 1 indicates perfect agreement. Two clinicians independently identified potential disease clusters which we compared to network analyses results. We found results differed greatly across combinations of association measures and cluster algorithms. The number of clusters identified ranged from 1 to 12 and their similarity was generally very low. Compared to clinician-derived clusters, the ARIs ranged from 0 to 0.23 indicating little similarity. These analyses demonstrate the need for a systematic evaluation of the performance of network analysis methods on binary clustered data like diseases. Moreover, diseases may not cluster, and a personalized approach to the care of older adults may be needed.

